# Ethnic Disparities in the Risk Factors, Morbidity, and Mortality of Cardiovascular Disease in People With Diabetes

**DOI:** 10.1210/jendso/bvae116

**Published:** 2024-06-12

**Authors:** Lekshmi Nair, Peace Asuzu, Sam Dagogo-Jack

**Affiliations:** Department of Medicine, Division of Endocrinology, Diabetes and Metabolism, University of Tennessee Health Science Center, Memphis, TN 38163, USA; Department of Medicine, Division of Endocrinology, Diabetes and Metabolism, University of Tennessee Health Science Center, Memphis, TN 38163, USA; Department of Medicine, Division of Endocrinology, Diabetes and Metabolism, University of Tennessee Health Science Center, Memphis, TN 38163, USA

**Keywords:** macrovascular complications, heart disease, health disparities, race/ethnicity, social determinants of health

## Abstract

Cardiovascular disease (CVD) is the leading cause of death in people with diabetes. Compared with European Americans, African Americans have more favorable lipid profiles, as indicated by higher high-density lipoprotein cholesterol, lower triglycerides, and less dense low-density lipoprotein particles. The less atherogenic lipid profile translates to lower incidence and prevalence of CVD in African Americans with diabetes, despite higher rates of hypertension and obesity. However, African Americans with CVD experience worse clinical outcomes, including higher mortality, compared with European Americans. This mini-review summarizes the epidemiology, pathophysiology, mechanisms, and management of CVD in people with diabetes, focusing on possible factors underlying the “African American CVD paradox” (lower CVD incidence/prevalence but worse outcomes). Although the reasons for the disparities in CVD outcomes remain to be fully elucidated, we present a critical appraisal of the roles of suboptimal control of risk factors, inequities in care delivery, several biological factors, and psychosocial stress. We identify gaps in current knowledge and propose areas for future investigation.

According to the Centers for Disease Control and Prevention (CDC), 38.4 million Americans had diabetes in 2021 [1]. Ethnic/racial disparities have been reported in the prevalence of both type 1 and type 2 diabetes. Compared with White or European Americans, Black or African Americans have a lower prevalence of type 1 diabetes but a higher prevalence of type 2 diabetes [2]. The age-adjusted prevalence of self-reported diabetes was 11.7% in African Americans, 8.1% in Asian Americans, 7.0% in European Americans, and 11.8% in Hispanic Americans [2]. Diabetes increases the risk for cardiovascular disease (CVD). In a meta-analysis, the hazard ratio (HR) for coronary heart disease (CHD) [HR 2.00 (95% confidence interval [CI] 1.83-2.19)], coronary death [HR 2.31 (95% CI 2.05-2.60)], and myocardial infarction (MI) [HR 1.82 (95% CI 1.64-2.03)] were higher in people with diabetes compared with those without diabetes [3].

This mini-review discusses CVD in African Americans with diabetes, with a particular focus on disparities in pathophysiology, management, and clinical outcomes.

## Search Strategy

We performed literature searches of English-language articles in PubMed/MEDLINE, Embase, and the Cochrane library using the following search terms: diabetes, CVD, coronary artery disease, coronary heart disease, stroke, heart failure, CVD mortality, Black, African American, ethnicity, and race. We selected pertinent articles published mostly within the past 10 years (2014-2024) but did not exclude pivotal older papers. Studies were included if they assessed associations between diabetes and CVD in different ethnic groups. We supplemented our searches with the identification of additional articles from references of selected sources with a focus on high-quality and representative cross-sectional studies, prospective cohort studies, randomized trials, systematic reviews, and meta-analyses. We also reviewed current practice guidelines. We excluded studies that had no informative ethnic/racial content regarding the CVD or diabetes data presented.

## Pathophysiology of CVD

### Role of Glucose

**Figure 1.**
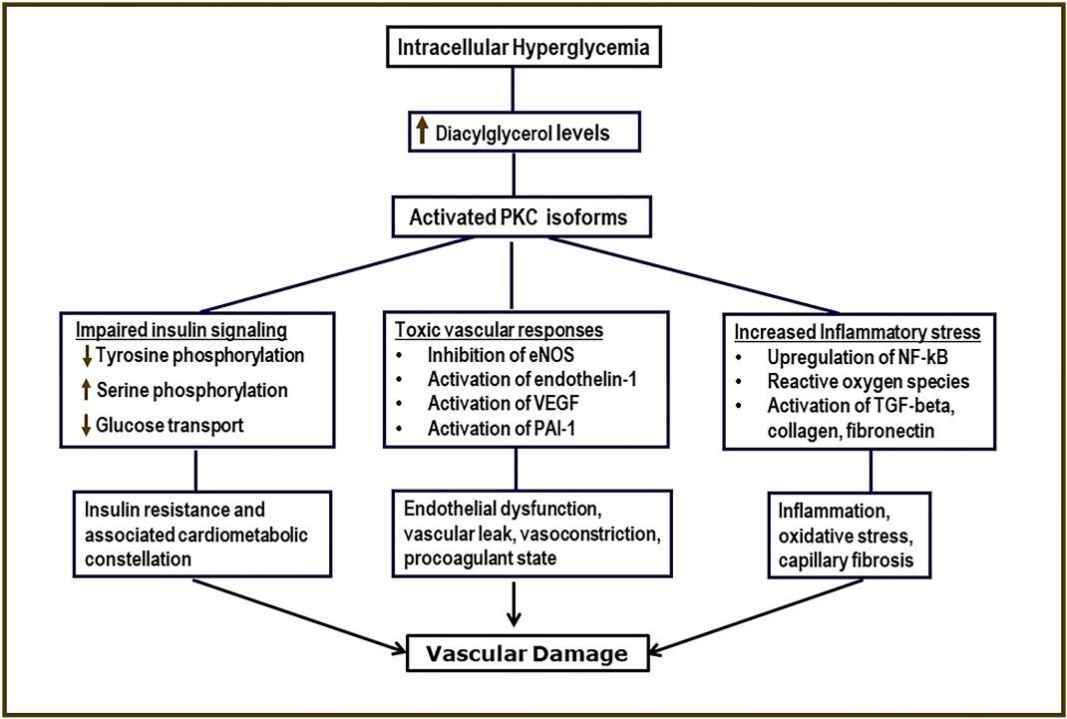
Mechanisms linking hyperglycemia to vascular complications mediated by activation of protein kinase C (see ref. 5).

Hyperglycemia is the primary cause of microvascular complications and one of multiple risk factors for macrovascular complications [4]. The mechanisms linking hyperglycemia to tissue damage involve multiple toxic pathways [5-7]. Intracellularly, excess glucose flux through the polyol pathway depletes antioxidant capacity and increases susceptibility to oxidative stress [5, 8, 9]. Hyperglycemia increases the production of advanced glycation end products, with consequent cell damage through modification of intracellular proteins, including those involved in gene transcription [5, 9, 10]. Intracellular glucose flux also increases the production of diacylglycerol; activation of protein kinase C isoforms; and modulation of genes involved in angiogenesis, vascular permeability, fibrosis, and inflammation (Fig. 1) [5, 9]. Additionally, increased glucose flux through the hexosamine pathway generates uridine diphosphate N-acetyl glucosamine and resultant gene modification [5, 8, 9]. Other factors, including insulin resistance, endothelial dysfunction, a pro-thrombotic state, and impaired fibrinolytic capacity, further contribute to CVD risks in people with diabetes [11, 12]. Studies have found poorer glycemic control in African Americans compared with European Americans with diabetes, which could contribute to disparities in vascular complications [13, 14].

### Traditional CVD Risk Factors

The traditional CVD risk factors in the general population, also evident in people with diabetes, include nonmodifiable elements (male sex, family history, older age) and modifiable factors (hypertension, dyslipidemia, smoking, obesity, and physical inactivity) [15, 16]. The prevalence of modifiable risk factors by race/ethnicity is discussed in the sections that follow.

#### Tobacco smoking

Smoking increases CVD risk via complex and interconnected mechanisms [17-19]. In a prospective study assessing modifiable CVD risk factors in 155 722 individuals recruited from 21 countries (including those from high-income, middle-income, and low-income regions), tobacco use was strongly associated with CVD events, with HRs and 95% CIs of 1.19 (CI 1.08, 1.31) and 1.64 (CI 1.51, 1.77) for former and current smokers, respectively [19]. In 2021, the CDC estimated that 11.5% (28.3 million) of US adults 18 years or older smoked cigarettes either daily or intermittently in addition to smoking ≥100 cigarettes during a lifetime [20]. The reported prevalence rate of current smokers in the United States is highest among European Americans (12.9%) followed by African Americans (11.7%), Hispanic Americans (7.7%), and Asian Americans (5.4%) [20].

#### Hypertension

Several studies have found hypertension to be the most significant modifiable CVD risk factor, accounting for 48% of all strokes and 18% of all coronary events [14, 16, 21, 22]. Approximately 46.7% of adults in the United States (122.4 million people) have hypertension, of whom more than one-third are unaware of having the disease [21]. The age-adjusted prevalence of hypertension among African American adults is 55.8% among men and 56.9% among women, compared with 46.6% and 40.0% in European American men and women, respectively [21]. Thus, African Americans have a prevalence of hypertension that is ∼1.3-fold higher than the rate among European Americans.

#### Dyslipidemia

Dyslipidemia, defined as elevated plasma total cholesterol, low-density lipoprotein (LDL) cholesterol or triglyceride levels, decreased plasma high-density lipoprotein (HDL) cholesterol level, or a combination of these patterns, is a major risk factor for atherosclerotic CVD [23-27]. Several studies have demonstrated the critical roles of LDL cholesterol and its oxidized form in the initiation and progression of atherosclerosis [23, 25-27]. Trends in serum total cholesterol levels among US adults from the National Health and Nutrition Examination Survey (NHANES) showed that African Americans had lower total cholesterol levels compared with European Americans and other groups during survey years 1999-2002, 2007-2010, and 2017-2020 [21]. African Americans have higher HDL cholesterol, lower triglycerides, and larger LDL particle size compared with other groups [28-30].

#### Overweight/abdominal obesity

From NHANES 2017-2020 data, the age-adjusted prevalence of overweight [body mass index (BMI) ≥ 25 and <30 kg/m^2^] was 71.2% and that of obesity (BMI ≥30 kg/m^2^) was 41.9% among adults ≥20 years of age in the United States [21]. Ethnic/racial differences have been observed in the prevalence of overweight, obesity, and abdominal obesity (using sex-specific cut-offs for waist circumference) [21, 31-36]. The BMI is limited as a measure of adiposity due to variability of bone mass, muscle mass, and other nonadipose soft tissue contributors to body weight [21, 32-34]. The BMI tends to overestimate obesity in African Americans and underestimate obesity in Asians [21, 34]. Waist circumference, a surrogate measure of visceral fat, is strongly associated with insulin resistance, CVD, and mortality risk [31, 34, 35]. African American women tend to have higher waist circumference than European American women, whereas African American men tend to have lower waist circumference than European American men [35-37].

#### Sedentary behavior

All forms of physical activity, regardless of age, sex, or ethnicity, have a beneficial effect on CVD prevention [21, 38, 39]. The benefits of physical activity include improved control of glycemia, blood pressure, and lipid profile; increased insulin sensitivity and cardiorespiratory fitness; and decreased mortality [21, 39]. Sedentary behavior is characterized by energy expenditure that is not higher than that achieved while seated or reclining during waking hours [38, 39]. Physical inactivity contributes to the progression of CVD and metabolic disorders [21, 38, 39]. Only 24.2% of adults in the United States self-reported meeting the guidelines for physical activity (ie, 150 minutes/week of moderate-intensity or ≥75 minutes/week of vigorous physical activity) [21, 38, 39]. Among men, the physical activity adherence rate was similar in African Americans, Asian Americans, and European Americans (29.7%, 30.2%, and 30.5%, respectively) and was 23.5% in Hispanic Americans [21]. Among women, the physical activity adherence rates were 16.5%, 24.3%, 18.0%, and 16.7% for African Americans, European Americans, Hispanic Americans, and Asian Americans, respectively [21]. These data indicate underachievement of the minimal physical activity targets across US demographic groups. This deficiency provides a unique opportunity for innovative interventions to increase physical activity.

#### Dietary habits

Healthy dietary habits are associated with general good health. For example, the Mediterranean-style eating pattern (based on consumption of fruits, vegetables, whole grains, seafood, legumes, nuts, and extra virgin olive oil) has been associated with healthy weight control and decreased risks of type 2 diabetes, CVD, and mortality [40-42]. In the case-control INTERHEART study, daily consumption of fruits and vegetables was one of 9 modifiable risk factors associated with incident MI [43]. The others were dyslipidemia, smoking, hypertension, diabetes, abdominal obesity, psychosocial factors, regular alcohol consumption, and regular physical activity. Physical inactivity and suboptimal diet accounted for 25.9% of the population-attributable risk associated with MI [43]. In the United States, suboptimal diet accounts for an estimated 45% of cardiometabolic deaths among adults each year: 53.1% among Black adults, 50.0% among Hispanic adults, and 42.8% among White adults [44]. High intake of sodium and sugar-sweetened beverages along with low intake of vegetables, fruits, nuts, seeds, and seafood accounted for most of the diet-related deaths [44].

Although unhealthy foods are heavily promoted in certain African American neighborhoods and are cheaper and more readily available than healthier alternatives, national surveys indicate broadly similar dietary patterns across the major US ethnic groups [45-47]. In most surveys, Black men and Black women reported lower consumption of saturated fat, vegetables, potassium, and calcium than White male and White female respondents [45-47]. In surveys confined to women from low socioeconomic status, non-Hispanic White women and non-Hispanic Black women reported lower consumption of vegetables, legumes, and whole grains, compared with non-Hispanic Asian American women and Mexican American women [48]. Rehm et al analyzed national secular trends in dietary habits, using the American Heart Association (AHA) diet score [47]. The AHA diet assessment comprises a primary score (maximum of 50 points) that tracks intake of total fruits and vegetables, whole grains, fish and shellfish, sugar-sweetened beverages, and sodium and a secondary score (maximum of 80 points) that tracks intake of nuts, seeds, legumes, processed meat, and saturated fat [47]. Between 2003 and 2012, the mean AHA primary diet scores improved only modestly from 19.3 to 21.7 in non-Hispanic White adults, from 17.2 to 18.9 in non-Hispanic Black adults, and from 17.5 to 18.3 in Mexican American adults [47]. The trends in AHA secondary diet scores also showed only modest to marginal improvements: from 35.1 to 38.8 in non-Hispanic White adults, from 31.8 to 34.8 in non-Hispanic Black adults, and from 36.5 to 36.8 in Mexican American adults. During the ∼10-year study period, intake of whole grains, nuts/seeds, and fish increased and consumption of sugar-sweetened beverages decreased, but there were no significant changes regarding consumption of fruits, vegetables, processed meat, saturated fat, or sodium [47]. Clearly, nonadherence to dietary recommendations is pervasive across the US population. Addressing this modifiable CVD risk factor should be a public health priority.

## Disparities in CVD Morbidity and Mortality

### Incidence and Prevalence of CVD

The risk for CVD is multiple-fold higher in people with diabetes vs those without diabetes [21, 49-51]. Besides disparities in the prevalence of diabetes, ethnic disparities manifest in the burden of diabetes complications [52-56]. Among 62 432 people with diabetes enrolled in the Kaiser Permanente Northern California health system, the prevalence of diabetic complications varied by ethnicity [53]. The adjusted HR [95% CI] (relative to European Americans) for MI was 0.56 [0.47-0.66] for African Americans, 0.68 [0.57-0.82] for Asian Americans, and 0.68 [0.56-0.81] for Hispanic Americans [53]. The adjusted HR [95% CI] (relative to European Americans) for end-stage kidney disease was 2.03 [1.62-2.54] for African Americans. No significant Black vs White differences were observed for stroke, heart failure, or lower extremity amputation in that insured population [53].

**Figure 2.**
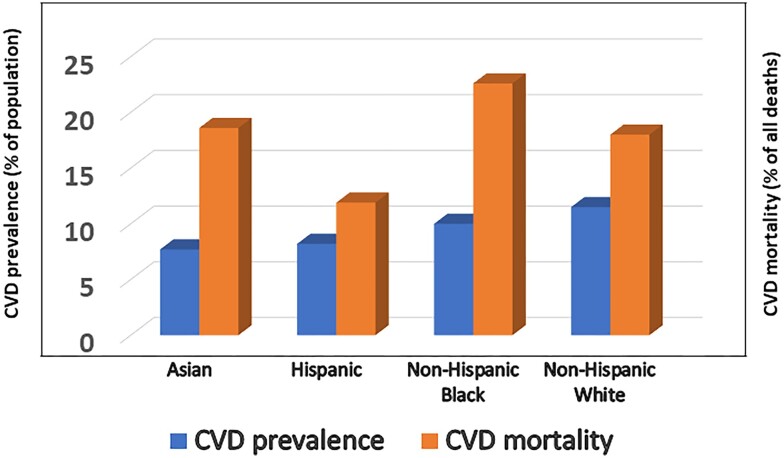
Cardiovascular disease prevalence and mortality by ethnicity (see refs. [21, 60]).

Similar findings were reported in a prospective study evaluating the 10-year risk of CHD by race/ethnicity, diabetes status, and prior CHD [55]. Analysis of data from 10 980 800 person-years of follow-up in the Kaiser Permanente Northern California health system showed that African Americans with diabetes had a lower incidence of CHD than European Americans with diabetes [55]. In population-wide data from NHANES 2017-2020, the age-adjusted prevalence of all heart disease was 11.5% among European Americans, 10.0% among African Americans, 8.2% among Hispanic Americans, 7.7% among Asian Americans, and 14.6% among American Indian or Alaska Native individuals [21]. These rates concord with data from another national survey showing CHD prevalence of 11.4% in European Americans, 10.0% in African Americans, 8.8% in Hispanic Americans, and 6.3% in Asian Americans and Pacific Islanders (Fig. 2) [56]. Thus, African Americans with or without diabetes have lower incidence and prevalence rates of CVD compared with European Americans. The lower CVD rates (despite the higher burden of hypertension, poorer glycemic control, and kidney disease) among African Americans vs European Americans could be due, in part, to a less atherogenic lipid profile [28-30].

### CVD Mortality and Secular Trends

Despite having consistently lower rates of CVD prevalence and incidence in cross-sectional and prospective studies, African Americans with CVD have worse clinical outcomes, including a disproportionately higher mortality rate (Fig. 2) [21, 57-65]. In a prospective study of 24 443 adults (10 348 Black, 14 095 White) without CHD at baseline, followed up for a mean of 4.2 years, incident nonfatal CHD events were lower in Black participants vs White participants [HR 0.68 (95% CI 0.51-0.91) for men and 0.81 (95% CI 0.58-1.15) for women] [58]. However, the age-adjusted incidence of fatal CHD was 2-fold higher in Black participants compared with White participants [58]. Interestingly, the 2-fold higher incidence of fatal CHD in Black vs White participants was markedly attenuated [HR 1.34 (95% CI 0.91-1.96) in men; 1.14 (95% CI 0.69-1.99) in women] after adjustments for lipids, smoking, blood pressure, diabetes, antihypertensive and statin medications, education level, and income [58].

Secular trends in CVD rates have shown a progressive decline during the past few decades, but the gradient has been smaller for African Americans compared with European Americans [59-63]. In the ARIC study, the secular decline in CHD incidence among African American men (−3.2%/year) was one-half that of the decrease in European American men (−6.5%/year), but the trends were similar in African American and European American women [59]. From the National Vital Statistics System data, a steady decrease in death rates among European Americans was seen during 1968-2000 [annual percentage change (APC) = 2.2%], followed by an accelerated decrease during 2000-2010 (APC = 3.9%) [63]. Among African Americans, the decline in death rates was initially slower than that of European Americans during 1968-1998 (APC 1.4% per year) but accelerated during 1998-2015 to an APC of 3.4% [63].

Jain et al used the CDC database to identify adults ≥25 years old where both CVD and diabetes were listed as the underlying or contributing cause of death between 1999 and 2019 [64]. The investigators found that the age-adjusted mortality rate was ∼2-fold higher in non-Hispanic Black adults compared with non-Hispanic White adults [64]. These data translate to a consistently higher African American/European American heart disease mortality rate ratio of ∼1.3 during the 1970s-1980s that has persisted and worsened over the ensuing decades [62-64]. During the peak of the COVID-19 pandemic, deaths from CVD increased in all ethnic groups in the United States compared to deaths during the immediate prepandemic year [65, 66]. Remarkably, the increase in COVID-19-associated CVD deaths was 3- to 5-fold higher in African Americans, Hispanic Americans, and Asian Americans compared with European Americans [65]. Thus, the COVID-19 pandemic exacerbated disparities in CVD that could persist well into the post-COVID-19 era [65-67].

## The African American CVD Paradox

### Dissociation Between Risk Factors and Outcomes

Compared with European Americans, African Americans generally have a healthier or less atherogenic plasma lipid profile, characterized by higher HDL cholesterol levels, lower triglycerides, and less dense LDL particles [28-30]. African Americans also have a higher frequency of loss-of-function mutations of the proprotein convertase subtilisin/kexin type 9 (PCSK9) gene, which is associated with lower levels of circulating PCSK9 and LDL cholesterol [68, 69]. Lower serum PCSK9 levels are protective of incident CVD, after adjustment for traditional risk factors [69]. Furthermore, African American men have lower visceral adiposity compared with European American men [35-37]. Among the other CVD risk factors, African Americans have higher prevalences of hypertension and type 2 diabetes and similar rates of active smoking compared with European Americans (Table 1). The partially beneficial CVD risk profile (less dyslipidemia) translates to lower incidence and prevalence rates of CVD in African Americans with diabetes compared with European Americans (Fig. 2) [53, 55, 56].

**Table 1.** Comparison of cardiovascular disease risk factors in African Americans and European American adults*^a^*

Risk factors	African American	European American
Cigarette smoking (%)	11.7	12.9
Abdominal obesity (%)		
Men	23.3	30.5
Women	44.6	37.2
Diabetes prevalence (%)	11.7	7.0
Hypertension prevalence (%)		
Men	57.6	46.7
Women	53.2	38.8
HDL cholesterol (mg/dL), mean (95% CI)	57.2 (56.5-57.9)	54.9 (54.3-55.5)
Total cholesterol (mg/dL), mean (95% CI)	186.2 (184.6-187.9)	191.6 (190.1-193.1)
LDL size, Å (± SEM)	262.1 ± 0.6	259.2 ± 0.4
Triglycerides (mg/dL), mean (95% CI)	89.2 (86.7-91.7)	117.4 (114.9-119.8)

Abbreviations: Å, Angstrom unit (1 Å = 10^−10^ m); CI, confidence interval; HDL, high-density lipoprotein; LDL, low-density lipoprotein.

^*a*^See references [20, 21, 29, 30, 35].

In contrast, African Americans with CVD experience worse clinical outcomes, including higher mortality, compared with European Americans (Fig. 2) [21, 57-67, 70]. What then can be responsible for the worse CVD outcomes in African Americans?

### Putative Mechanisms and Drivers of the African American CVD Paradox

As the traditional risk factors do not fully explain the poorer CVD outcomes in African Americans, studies have explored alternative explanations. What follows is an overview of some factors that provide additional insights [71].

#### Insulin resistance

It has been suggested that the favorable lipid profile in African Americans may not translate to superior CVD outcomes because of prevalent insulin resistance that counteracts the expected lipid benefits [72, 73]. However, Carnethon et al [74] observed that most traditional CVD risk factors exhibit similar associations with mortality across racial/ethnic groups. Insulin resistance is, indeed, an underlying feature of the metabolic syndrome, with broad associations with cardiometabolic risk markers. Cross-sectional studies have reported greater insulin resistance in African Americans and individuals from other ethnic groups compared with European Americans [75]. However, studies that targeted insulin resistance with specific pharmacological agents did not show compelling data regarding CVD risk reduction in people with type 2 diabetes [76, 77].

A placebo-controlled study targeting insulin resistance with pioglitazone in people with a prior history of stroke or transient ischemic attack did show significant reductions in the primary outcome of recurrent stroke or MI [78]. The HR for risk reduction (0.77 in White, 0.67 in Black, and 0.58 in Hispanic participants) showed robust efficacy of pioglitazone in the presumably more insulin-resistant Black and Hispanic participants [78].

#### Psychosocial stress

High psychosocial stress is a proposed mechanism for adverse health outcomes in African Africans compared with European Americans [79]. In the INTERHEART study, psychosocial stress exerted a higher attributable risk for MI than did hypertension and diabetes combined [43]. The extent to which increased psychosocial stress burden contributes to clinical outcomes in African Americans with CVD is not fully understood. Carefully designed studies, with rigorous assessment of stressors and stress levels along with robust clinical outcome measures, could be illuminating [80].

#### Coronary artery calcium

Coronary artery calcium (CAC) score, a marker of atherosclerosis that augments the accuracy of CVD risk stratification, has been examined in multiethnic populations [81-86]. The reports indicate that African Americans have lower CAC scores than European Americans, after adjustments for traditional CVD risk factors [85, 86]. Furthermore, African Americans have 50% lower odds of having a significant CAC score compared with European Americans [85]. To the extent that higher CAC scores have negative connotations for CVD health, the lower scores of African Americans do not provide a mechanism for the higher CVD mortality in that population.

#### HDL functionality

The higher CVD mortality observed among African Americans is on a background of the usually higher levels of HDL cholesterol in African Americans [28, 29]. Because HDL has antiatherogenic and cardioprotective properties, its functionality has been assessed as a potential explanation for the African American CVD paradox. Paraoxonase-1 (PON1), an HDL-associated enzyme with antioxidant functions, exerts cardioprotective effects by protecting LDL cholesterol from oxidation [87-92]. Mice with PON1 gene deletion develop atherosclerosis faster than control mice on a high-fat, high-cholesterol diet [87]; overexpression of PON1 in transgenic mice decreases atherosclerosis risk [87, 89]. In human beings, an inverse relationship between serum PON1 activity and CVD risk has been reported [88, 90-94]. Thus, theoretically, the generally higher HDL cholesterol levels observed in African Americans, if associated with reduced PON1 activity, could be less functional and consequently less cardioprotective. However, serum PON1 levels were reported to be similar (0.6 ± 0.3 ng/dL vs 0.6 ± 0.3 ng/dL) in African Americans vs European Americans with prediabetes [94]. Among healthy subjects aged 25 to 65 years, African Americans had higher serum PON1 activity compared with European Americans (297 ± 144 units vs 183 ± 121 units, *P* < 0.05) [95]. Thus, dysfunctional HDL due to suboptimal PON1 activity is not a likely contributor to the adverse CVD outcomes in African Americans.

Cholesterol efflux capacity (CEC) is another measure of HDL functionality that has been examined across ethnic groups [96-98]. The Dallas Heart Study investigators reported that, compared with other participants, Black participants had significantly higher serum HDL cholesterol levels (median [interquartile range] 49 [42-60] mg/dL vs 45 [38-55] mg/dL) and marginally but significantly lower CEC (median [interquartile range] 0.98 [0.81-1.18]) vs 1.01 [0.85-1.19]) [96]. A subsequent report from the Dallas Heart Study found that low CEC was associated with younger age, male sex, and Black race when measured with a radiolabeled technique, but those associations were not seen when CEC was measured with a fluorescent technique [97]. In a different multiethnic study, cholesterol efflux capacity (measured with a fluorescent technique) was inversely associated with self-reported Black status, male sex, higher estimated glomerular filtration rate, and use of statin drugs [98].

As an emerging nontraditional CVD risk factor, several studies have reported an association between greater CEC and lower risk of CVD [96, 97, 99-101]. Thus, the reported association between low CEC and Black ancestry deserves further investigation.

#### Inflammatory markers

African Americans tend to have higher levels of the proinflammatory C-reactive protein and lower levels of the anti-inflammatory adiponectin compared with European Americans [21, 102-105]. In the prospective JUPITER study, pharmacological intervention with rosuvastatin (vs placebo) to target inflammation (based on C-reactive protein levels) significantly decreased the combined outcome of MI, stroke, or death from CVD causes [HR 0.53 (95% CI 0.40-0.69)] [106]. Clearly, inflammation is a modifiable risk factor for poor CVD outcomes and a potential factor underlying ethnic disparities in CVD outcomes [106, 107]. Despite the impressive results from the JUPITER study, risk stratification and treatment based on C-reactive protein levels is not a widespread practice. Such a practice could inform a more comprehensive cardioprotective coverage for African Americans, given their relatively higher C-reactive protein levels. National surveys indicate that statin therapy is underutilized in African American and Hispanic American patients [70].

#### Platelet function

Coagulation, thrombosis, and fibrinolysis are critical determinants of occlusive vascular events, such as stroke and MI. Platelet activation is vital to the formation of an occlusive thrombus. Interindividual and ethnic variations in platelet reactivity have been reported [108-111]. Thrombin initiates the thrombotic cascade through platelet activation by signaling via protease-activated receptor (PAR) 1 or PAR4, expressed by human platelets. In a study comparing platelet function, prothrombotic stimuli that signal via PAR1 (eg, arachidonic acid, adenosine diphosphate, anti-CD9 antibody, and collagen-related peptide) triggered similar maximal platelet aggregation responses in African Americans and European Americans [108]. However, activation through the PAR4 thrombin receptor resulted in aggregation responses that were 3.8-fold higher in platelets from African Americans compared with the responses in platelets from European Americans [108]. The differences in platelet aggregation were not attenuated by cyclooxygenase and adenosine diphosphate-P2Y12 dual inhibition, suggesting that current antiplatelet therapies may provide less adequate protection for African Americans compared with European Americans [110, 111]. Further studies in this area could potentially lead to the development of novel antiplatelet therapies to reduce disparities in CVD outcomes.

#### Sleep pathologies

Sleep disorders have been linked to a plethora of negative consequences, including increased risks for cardiometabolic, neurodegenerative, and inflammatory disorders [112, 113]. The American Academy of Sleep Medicine and the Sleep Research Society recommend that adults obtain at least 7 hours of sleep per night to promote optimal health [114]. In national surveys of adults, a greater proportion of African Americans report having suboptimal sleeping duration (<7 hour/night) compared with European Americans [21, 115]. The Jackson Heart Study sleep study reported that African American adults had a 61.5% prevalence of actigraphy-measured short sleep duration [116]. In another study, African American participants were approximately 5 times more likely to experience actigraphy-measured short sleep duration (<6 hours) compared with European American participants [117]. Thus, it is plausible that disparities in sleep parameters could contribute to ethnic disparities in CVD outcomes [21, 113-121].

In summary, the foregoing consideration of nontraditional CVD risk factors has identified low cholesterol efflux capacity, inflammation, suboptimal sleep duration, and platelet hyperreactivity as putative contributors to adverse CVD outcomes in African Americans. These elements are veritable targets for carefully designed future studies aimed at dissecting their mechanisms and developing interventions to reduce disparities in CVD outcomes.

## Management of Cardiovascular Disease

### Disparities in Control of CVD Risk Factors

Suboptimal glycemic control in African American patients suggests treatment inertia or lack of guidelines-directed intensification of antidiabetes therapy [122, 123]. Among individuals with Medicare Advantage health plans, African Americans achieved significantly less optimal control of blood pressure (adjusted difference, 10.3% points), LDL cholesterol (10.2% points), and hemoglobin A1c (9.4% points) compared with European Americans [124]. Matching African American and European American enrollees by health plans reduced the relative differences by 39% to 59%, indicating that African Americans were disproportionately enrolled in lower-performing health plans [124]. Examined by regions of the United States, disparities in the control of CVD risk factors disappeared over time in the West but persisted in the Northeast, Midwest, and South [124]. The improvements in the West show that equity in health outcomes is achievable [124]. Thus, the persisting inequities should be of concern to clinicians [70].

### Disparities in Management of CVD Events

Following CVD events, racial/ethnic differences in quality of care have been reported across a broad spectrum of areas [125-131]. Data from 397 257 patients admitted with ischemic stroke between 2003 and 2008 showed that African American patients had lower odds of receiving intravenous thrombolysis, deep vein thrombosis prophylaxis, smoking cessation counseling, anticoagulants for atrial fibrillation, and lipid-lowering therapy compared with European American patients [126]. Similar suboptimal care for African Americans has been documented regarding thresholds for cardiac catheterization, percutaneous coronary intervention, use of drug eluting stents, and revascularization procedures [126-130]. Furthermore, African Americans hospitalized with MI experience iatrogenic delays in time to acute reperfusion therapy [131]. These systemic inequities in quality and timeliness of care could explain, at least in part, the conundrum of lower prevalence/incidence of CVD, yet higher CVD deaths, in African Americans compared with European Americans.

### Cardiovascular Outcomes Trials

Macrovascular complications, including CVD, account predominantly for deaths in people with diabetes. Thus, the results of clinical trials of antidiabetes medications on CVD outcomes in people with type 2 diabetes are of particular interest to clinicians [132-138]. Collectively, these cardiovascular outcomes trials (CVOTs) have demonstrated safety for the major classes of antidiabetes agents in use and risk reductions for adverse CVD and kidney outcomes with medications from the sodium glucose co-transporter (SGLT)-2 inhibitor and the glucagon-like peptide−1 receptor agonist (GLP-1ra) classes. The studies with SGLT-2 inhibitors showed a variable 3% to 14% relative risk reductions for major adverse cardiovascular events (MACE) and a consistent ∼30% relative risk reductions for hospitalization for heart failure across individual drugs [132-135]. The studies with GLP-1ras showed 12% or greater relative risk reductions for MACE and a neutral effect on hospitalization for heart failure [136-138]. Reductions in the risk of stroke were also reported with some of these medications [137, 138]. These data from the CVOTs have led to updated guidelines that prioritize the use of medications from the SGLT-2 inhibitor and GLP-1ra classes in diabetes patients, to decrease the risks of atherosclerotic CVD, hospitalization for heart failure, and progression of kidney disease [139].

The overall population enrolled in the CVOTs lacked diversity [140, 141]. In a meta-analysis of 19 randomized controlled trials that reported CVD outcomes, the total population of 136 178 participants comprised only 4.35% who were African American or Black and 10% who were of Hispanic, Pacific Island, Hawaiian, American Indian, and Alaska Native origin [140]. Most participants in those studies self-identified as White (68.1%) or Asian (17.9%) [140]. Using a random effects model, Cai et al calculated that the odds ratio (OR) for overall primary CVD outcomes was 0.74 [95% CI 0.65-0.84] in Asian participants, 0.84 [95% CI 0.62-1.14] in Black participants, 0.88 [95% CI 0.83-0.94) in White participants, and 0.85 [95% CI 0.73-0.99] in the combined enrollees from Hispanic, Pacific Islander, Hawaiian, American Indian, and Alaska Native ethnicities [140]. The point estimate for risk reduction overall was consistent across race/ethnicity; however, the lack of statistically significant risk reduction due to wide CI in Black patients probably reflects small sample size. However, a meta-analysis of the 15 placebo-controlled studies that prespecified composite primary outcome of MACE showed no significant risk reduction in Black participants (Table 2) [140]. In a meta-analysis confined to studies that tested GLP-1ras vs placebo, Cai et al observed no significant CVD risk reduction among Black participants [OR 1.08 (95% CI 0.62-1.90)], whereas benefits were observed in Asian participants [OR 0.66 (95% CI 0.54-0.83)] and White participants [OR 0.87 (95% CI 0.80-0.94)] (Table 3) [140]. In contrast, a meta-analysis confined to studies that tested SGLT-2 inhibitors vs placebo showed significant CVD risk reductions in African Americans [OR 0.60 (95% CI 0.43-0.82)] and other participants (Table 4) [140]. Like the findings by Cai et al [140], other workers have reported ethnic/racial disparities in risk reduction with SGLT-2 inhibitors and GLP-1ras in the CVOTs [141-143].

**Table 2.** Odds of major adverse cardiovascular events in a meta-analysis of 15 randomized placebo-controlled trials

Ethnic group	Number of participants (active/control)	Proportion (%)	Odds ratio	95% confidence interval
Asian	10 409/9788	16.96	0.80	0.66-0.85
Black	2518/2508	4.22	1.00	0.70-1.41
White	42 067/39 507	68.52	0.87	0.81-0.93
Others^*a*^	6197/6063	10.30	0.91	0.79-1.05

Adapted from Cai et al [140].

^*a*^People of Hispanic, Pacific Island, Hawaiian, American Indian, Alaska Native origins.

**Table 3.** Odds of primary cardiovascular outcomes in a meta-analysis of randomized placebo-controlled trials using GLP-1 receptor agonists as active agents

Ethnic group	Number of participants (active/control)	Proportion (%)	Odds ratio	95% confidence interval
Asian	2101/2130	8.47	0.66	0.54-0.83
Black	1120/1173	4.59	1.08	0.62-1.90
White	18 203/18 239	72.98	0.87	0.80-0.94
Others*^a^*	3517/3448	13.95	0.86	0.71-1.03

Adapted from Cai et al [140].

Abbreviation: GLP-1, glucagon-like peptide 1.

^*a*^People of Hispanic, Pacific Island, Hawaiian, American Indian, Alaska Native origins.

**Table 4.** Odds of primary cardiovascular outcomes in a meta-analysis of randomized placebo-controlled trials using SGLT2i inhibitors as active agents

Ethnic group	Number of participants (active/control)	Proportion (%)	Odds ratio	95% confidence interval
Asian	4498/3834	18.71	0.68	0.59-0.80
Black	627/506	2.54	0.60	0.43-0.82
White	17 261/14 808	72.02	0.82	0.71-0.94
Others*^a^*	1523/1468	6.72	0.68	0.52-0.90

Adapted from Cai et al [140].

Abbreviation: SGLT, sodium glucose co-transporter.

^*a*^People of Hispanic, Pacific Island, Hawaiian, American Indian, Alaska Native origins.

As the CVOTs did not prespecify comparison of results by race/ethnicity and were not powered for that purpose, the post hoc findings of apparent ethnic disparities in outcomes must be interpreted with caution [140-143]. Nonetheless, those findings stress the need for adequate representation in clinical trials to ensure that findings are generalizable across demographic groups. The risks of stroke, kidney disease, and heart failure are greater in people with diabetes than those without diabetes and higher in African Americans compared with European Americans [21, 144-147]. Thus, the reported benefits of GLP1-ras and SGLT-2 inhibitors on stroke, heart failure, and kidney disease are particularly desirable in African Americans with diabetes and need to be demonstrated definitively in that population [148].

### Approach to CVD Risk Reduction in African Americans With Diabetes

The approach to the management of CVD is summarized in Table 5. Educating patients regarding the comorbid risk factors (hypertension, hyperglycemia, dyslipidemia, etc.) is critical for successful shared decision-making. Initial conversations should emphasize that successful outcomes depend on the individual patient assuming a locus of control regarding the implementation of treatment recommendations. Given the high prevalence of obesity, the well-known benefits of lifestyle modification on blood pressure, glucose, lipids, weight control, and cardiovascular health must be repeatedly emphasized [149-152]. Lifestyle modification is the foundational therapeutic platform upon which selected medications are added as needed. Clinicians should stress to patients that the introduction of medications does not signal the abandonment of healthy lifestyle practices. Consonant with educating patients and promoting adherence behavior is the need for achieving recommended targets for control of risk factors (including blood pressure, diabetes, dyslipidemia, overweight/obesity, sleep impairment) [139, 149-153]. Most importantly, healthcare should be delivered in a manner that is equitable and devoid of overt or implicit biases [154-159].

**Table 5.** Approach to cardiovascular disease risk reduction in African Americans with diabetes

Address lifestyle factors	Optimize control of risk factors	Address psychological factors
Dietary modification	Blood pressure	Patient-centered care
Physical activity	Blood glucose	Health literacy/locus of control
Avoid overweight/obesity	Dyslipidemia	Shared decision-making
Sleep hygiene	Evidence-based targets	Advocacy for access to care
Smoking cessation	Evidence-based therapies	Equity in care delivery
Stress management	Primary/secondary prevention	Subconscious bias Social determinants of health

## Conclusions and Future Directions

African Americans have a lower incidence and prevalence of CVD but higher CVD mortality compared with European Americans. The reasons for the disparities in CVD outcomes are not fully understood, but evidence points to suboptimal control of risk factors and unequal care as possible contributors [124-131]. Additionally, a proinflammatory milieu, platelet hyperreactivity, and possibly cholesterol efflux capacity are putative biological factors underlying disparities in CVD outcomes [96-98, 103-111]. Further studies are needed to unravel the full spectrum of biological and psychosocial determinants of the dissociation between CVD incidence and CVD outcomes in African Americans. Potential targets for such research include a detailed evaluation of the roles of inflammation, oxidative stress markers, free radicals, antioxidant capacity, psychosocial stress, sleep, chronobiology, and circadian disruption [113-119, 158, 159]. Additional explanatory insights potentially could emerge from an evaluation of endothelial progenitor cells and genomic, transcriptomic, proteomic, and epigenetic mechanisms. Further areas of investigation pertinent to disparities in CVD outcomes include the roles of geocoding and the external built environment, exposure to air pollution and particulate matter, and the microbiome and metagenome, among other plausible mediators [160] (Table 6).

**Table 6.** Potential areas of future research to unravel mechanisms underlying disparities in cardiovascular disease outcomes

External/internal environments	Inflammation/oxidative stress
Geocoding/built environment	Pro-/anti-inflammatory markers
Air pollution/particulate matter exposure	Antioxidant capacity (superoxide dismutase)
Social determinants of health	HDL functionality
The microbiome and metagenome	Endothelial progenitor cells
**Coagulation/fibrinolysis**	**Chronobiology**
Platelet reactivity	Psychosocial stress
Fibrinolysis markers	Sleep pathologies
Pharmacogenetics of antiplatelet drugs	Endocrine disruption
**Molecular cardiology**	
Genomic, transcriptomic, proteomic, and epigenetic mechanisms	
Gene × Lifestyle interactions	

Abbreviation: HDL, high-density lipoprotein.

## Data Availability

Data sharing is not applicable to this article as no data sets were generated or analyzed during the current study.
